# The overlap of accessory virulence factors and multidrug resistance among clinical and surveillance *Klebsiella pneumoniae* isolates from a neonatal intensive care unit in Nepal: a single-centre experience in a resource-limited setting

**DOI:** 10.1186/s41182-024-00595-3

**Published:** 2024-04-08

**Authors:** Raj Kumar Shrestha, Dhruba Shrestha, Ajaya Jang Kunwar, Sandeep Thapa, Nipun Shrestha, Bhim Gopal Dhoubhadel, Christopher M. Parry

**Affiliations:** 1 Siddhi Memorial Hospital, Bhimsensthan-7, Bhaktapur, Nepal; 2 Kathmandu Center for Genomics and Research Laboratory, Lalitpur, Nepal; 3 School of Tropical Medicine and Global Health (TMGH), Nagasaki University, Nagasaki, 852-8523 Japan; 4 Department of Respiratory Infections, Institute of Tropical Medicine, Nagasaki University, Nagasaki, Japan; 5 Clinical Sciences and Education, Liverpool School of Tropical Medicine, Liverpool, UK

**Keywords:** Extended-spectrum β-lactamase, *Klebsiella pneumoniae*, Neonatal intensive care unit (NICU), Nepal, Virulence

## Abstract

**Background:**

There is a lack of data on the characteristics of overlap between acquired antimicrobial resistance and virulence factors in *Klebsiella pneumoniae* in high-risk settings, especially with the inclusion of surveillance isolates along with the clinical. We investigated *K. pneumoniae* isolates, from a neonatal intensive care unit (NICU) in Nepal, for the presence of both accessory virulence factors and acquired antimicrobial resistance.

**Methods:**

Thirty-eight clinical and nineteen surveillance *K. pneumoniae* isolates obtained between January 2017 and August 2022 in the NICU of Siddhi Memorial Hospital, Bhaktapur, Nepal were investigated with antimicrobial susceptibility testing, PCR-based detection of β-lactamases and virulence factors, and genetic similarity by ERIC–PCR.

**Results:**

*K. pneumoniae* was found positive in 37/85 (43.5%) blood culture-positive neonatal bloodstream infections, 34/954 (3.6%) patient surveillance cultures, and 15/451 (3.3%) environmental surveillance samples. Among 57 isolates analyzed in this study, we detected multidrug resistance in 37/57 (64.9%), which was combined with at least one accessory virulence factor in 21/37 (56.8%). This overlap was mostly among β-lactamase producing isolates with accessory mechanisms of iron acquisition. These isolates displayed heterogenous ERIC–PCR patterns suggesting genetic diversity.

**Conclusions:**

The clinical significance of this overlap between acquired antimicrobial resistance and accessory virulence genes in *K. pneumoniae* needs further investigation. Better resource allocation is necessary to strengthen infection prevention and control interventions in resource-limited settings.

**Supplementary Information:**

The online version contains supplementary material available at 10.1186/s41182-024-00595-3.

## Introduction

Acquisition of accessory virulence factors in addition to core virulence can enhance the pathogenic potential of *Klebsiella pneumoniae* infections [1]. Core virulence factors include an enterobactin siderophore, type 1 and 3 fimbriae, and two surface polysaccharides, surface capsule and lipopolysaccharide, encoded by *ent*, *fim*, *mrk*, K locus, and O locus, respectively [1]. *Kfu* (ferric iron uptake system), *alls* (allantoin metabolism), *clb* (colibactin, a genotoxic polyketide), and three siderophores, *iuc* (aerobactin), *ybtS* (yersiniabactin), and *iro* (salmochelin), are accessory virulence factors [1].

The overlap of extended-spectrum β-lactamases (ESBLs) and carbapenemases with two accessory virulence factors, aerobactin and yersiniabactin, was previously demonstrated in Asia among clinical isolates [2]. Analysis of such overlap, with the inclusion of surveillance isolates, is lacking in high-risk settings, such as neonatal intensive care unit (NICU). We aimed to investigate the occurrence of strains harbouring accessory virulence factors and displaying multidrug resistance (MDR) among clinical and surveillance isolates in a NICU in Nepal.

## Materials and methods

We investigated a convenience sample of 57 *Klebsiella pneumoniae* isolates (38 clinical, 12 surveillance, and 7 environmental) obtained between January 2017 and August 2022 in the NICU of Siddhi Memorial Hospital, a 50-bedded secondary care pediatric hospital. Antimicrobial susceptibility testing was performed by Kirby Bauer disk diffusion and interpreted according to CLSI guidelines (32nd edition) [3]. Isolates with non-susceptibility to any β-lactams were subjected to the D72C test (MAST, UK). A modified carbapenem inactivation method confirmed carbapenemase production among those suspected by the D72C test [3]. Multidrug resistance (MDR) was defined as non-susceptibility to one or more antimicrobials of three or more different antimicrobial classes [4].

Based on the phenotypic results, isolates were screened using PCR assays for extended-spectrum β-lactamases (ESBLs) (*bla*_CTX-M_, *bla*_TEM_) [5, 6], plasmid-mediated AmpC β-lactamases (pAmpC) (*bla*_MOX_, *bla*_CIT_, *bla*_DHA_*, bla*_ACC_*, bla*_EBC,_ and *bla*_FOX_) [7], and carbapenemases (*bla*_OXA-48_, *bla*_NDM_, *bla*_KPC_*, bla*_IMP_*,* and *bla*_VIM_) [8]. PCR was used to screen for seven virulence factors (*entB*, *mrkD*, *kfu*, *allS*, *iutA*, *ybtS*, *rmpA*), K1/K2 capsular serotypes, and *peg-344* gene [9, 10]. Genetic similarity between the isolates was determined by ERIC–PCR [11]. ERIC–PCR fingerprints were analysed using GelJ software (version 2.0) [12]. Dendrogram was generated by the Dice similarity method and UPGMA linkage. The similarity of > 90% was considered for genetic similarity. The laboratory methods can be found in supplementary data. The isolate data and the clinical data were acquired from the laboratory hospital record book and the medical records of the hospital, respectively.

## Results

**Fig. 1:**
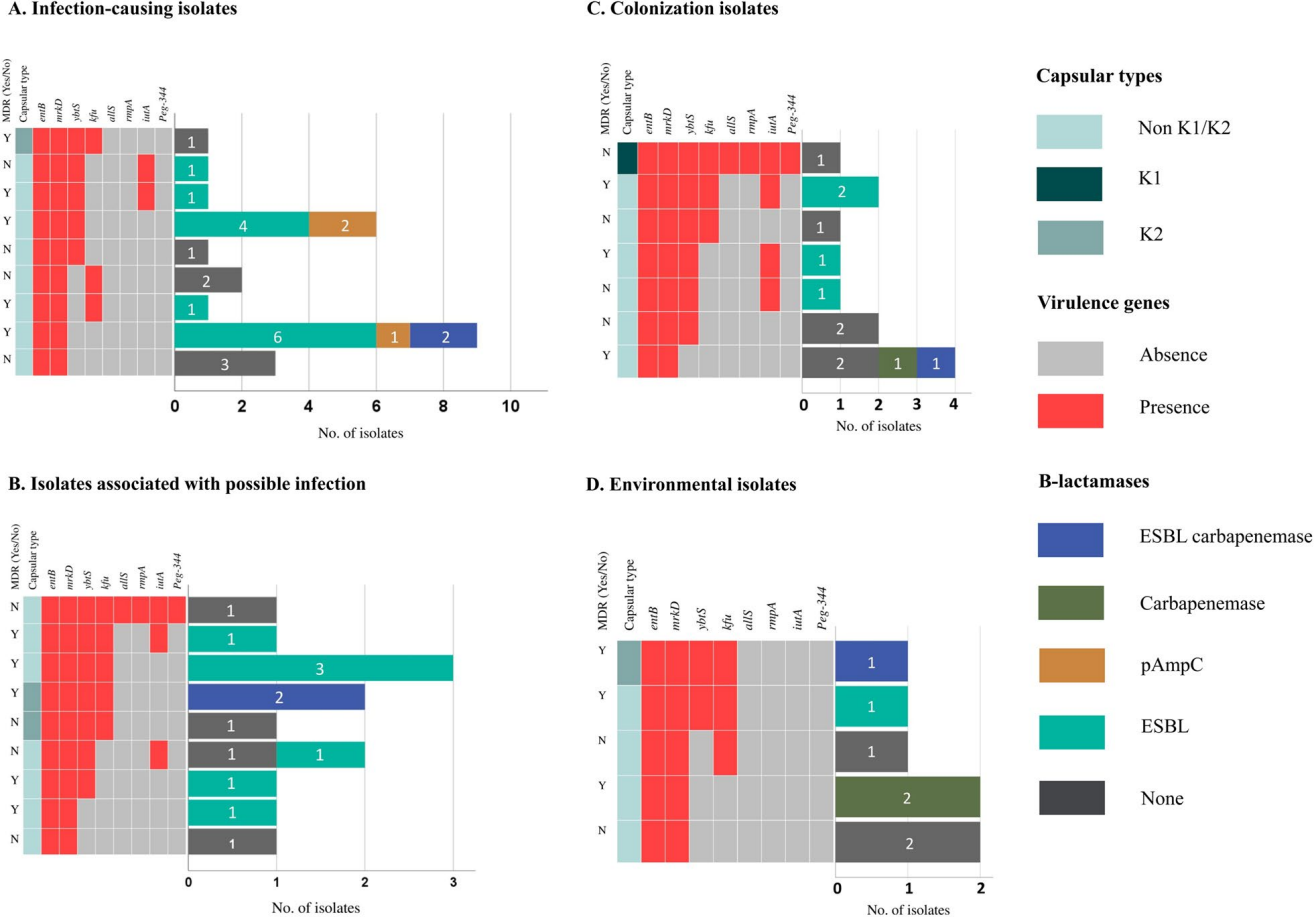
Heatmap showing the presence of β-lactamase genes and virulence factors and multidrug-resistant status stratified by **A** infection-causing isolates, **B** isolates associated with possible infection, **C** colonization isolates, and **D** environmental isolates. Infection-causing isolates include bloodstream infection-causing isolates (*n* = 24) and one isolate from pus. Possible infection-causing isolates are isolates from ET tube culture (*n* = 10) and UVC tip culture (*n* = 3). Colonizing isolates include those obtained from cultures of armpit swab (*n* = 1), rectal swabs (*n* = 2), and umbilical swabs (*n* = 9). Environmental isolates were obtained from the basin (*n* = 5) and patient’s bed surface (*n* = 2) swab cultures

Within the study duration, 37/85 (43.5%) culture-proven bloodstream infections among the neonates attending Siddhi Memorial Hospital were attributed to *K. pneumoniae*. *K. pneumoniae* was found positive in 34/954 (3.6%) patient surveillance and 15/451 (3.3%) environmental surveillance samples (Additional file 1: Fig. S1). The proportions of non-susceptibilities to the antimicrobials are presented in Additional file 4: Table S1. Among the 57 isolates, non-susceptible to: piperacillin–tazobactam was 20 (35.1%), extended-spectrum cephalosporins was 42 (73.7%), meropenem was 11 (19.3%), ciprofloxacin was 35 (61.4%), and amikacin was 15 (26.3%). Non-susceptibility to extended-spectrum cephalosporins was attributed to 25 ESBL, 3 pAmpC, 3 carbapenemase, 6 ESBL/carbapenemase co-producers, and two β-lactamase negatives (Fig. 1 and Additional file 5: Table S2). Of 57 isolates, 37 (64.9%) were MDR.

Accessory virulence genes were detected in 35/57 (61.4%) isolates and ranged from one to five genes (Fig. 1 and Additional file 5: Table S2). A total of 21/37 (56.8%) MDR isolates had at least one accessory virulence factor. This overlap was mostly among β-lactamase producing isolates with accessory mechanisms of iron acquisition. Among those 21 MDR isolates with the overlap, one had *kfu* and produced ESBL, 9 possessed *ybtS* (seven ESBL and two pAmpC producers), and another 10 had both *ybtS* and *kfu* (seven ESBL and three ESBL/carbapenemase co-producers) along with other virulence factors.

Two isolates displayed hypermucoid phenotype and possessed *rmpA*, *iutA*, and *peg-344* (features considered consistent with hypervirulence) along with *ybtS*, *alls*, and *kfu* and were likely hypervirulent *K. pneumoniae* (HvKp). These two HvKp isolates did not have acquired resistance. Nine additional isolates were positive for *iutA* suggesting suspected hypervirulence, but none of these isolates showed hypermucoid phenotype. Of 11 *iutA* positive strains, 8 isolates had both *bla*_CTX-M_ and *bla*_TEM_ and 5 of them were MDR. The ERIC–PCR analysis suggested genetic diversity among the isolates (Additional file 3: Fig. S3).

## Discussion

Our study documents an overlap between MDR and accessory virulence factors in *K. pneumoniae* from a NICU in Nepal. This overlap was most common with acquired mechanisms of iron acquisition and β-lactamase production, commonly ESBL. Yersiniabactin siderophore (*ybtS*), the most common acquired virulence factor overall, was frequent among the isolates with the overlap. Yersiniabactin was previously shown to be significantly associated with ESBL and carbapenemase in an analysis of invasive *K. pneumoniae* from seven Asian countries [2], but not in Australia [13]. Studies indicate the potential for *ybt* harbouring strains to progress from a colonizing niche to infection [14] and to cause outbreaks of systemic infections among hospitalized children [15]. The unavailability of complete clinical data among the neonatal infections in our study precluded comparison of clinical aspects.

The ERIC–PCR analysis suggests that the overlap between MDR and accessory virulence is less likely to be attributed to genetically similar strains. This observation is consistent with a study from Australia suggesting that the burden of *K. pneumoniae* infections among hospitalized patients is largely attributed to opportunistic infections with genetically diverse strains [13]. We found two potential HvKp isolates and nine additional suspected HvKp [1]. HvKp invasive infections may be associated with a high mortality [1]. Their prompt detection in high-risk settings, such as NICU, to contain their spread requires laboratory capacity.

Our study demonstrates an overlap between accessory mechanisms of iron acquisition and β-lactamases, in genetically heterogeneous strains. Future studies should explore differences in clinical outcomes among *K. pneumoniae* infections with and without the overlap of AMR and accessory virulence, especially yersiniabactin. Better resource allocation is necessary to strengthen infection prevention and control interventions in resource-limited settings.

## Limitations

In this study, the total number of isolates analyzed were limited. Investigations with larger sets of isolates from both secondary and tertiary care level healthcare settings will be helpful. Such inclusion of more healthcare centres is necessary to extend the generalizability of the findings reported here.

### Supplementary Information

**Additional file 1: Figure S1**. Results of A) neonate surveillance swabs and B) environmental surveillance swab cultures at the NICU of SMH within the study duration showing *K. pneumoniae* as one of the commonly isolated pathogens.

**Additional file 2: Figure S2**. Gel electrophoresis results of PCR amplification products of representative samples showing genes investigated in this study.

**Additional file 3: Figure S3.** Dendrogram of ERIC–PCR fingerprints of 57 K*. pneumoniae* isolates based on Dice similarity and UPGMA linkage showing significant genetic heterogeneity among the isolates.

**Additional file 4: Table S1**. Antimicrobial non-susceptibility patterns of infection-causing, possibly infection-causing, colonizing, and environmental *K. pneumoniae* isolates.

**Additional file 5: Table S2**. Distribution of β-lactamases stratified number of accessory virulence genes among infection-causing, colonizing, and environmental isolates.

**Additional file 6: Text**. Additional details on the laboratory methods.

## Data Availability

All data generated or analysed during this study are included in this published article and its additional information files.

## Abbreviations

ESBL: Extended-spectrum β-lactamase
MDR: Multidrug resistance
NICU: Neonatal intensive care unit
CLSI: Clinical and Laboratory Standards Institute
PCR: Polymerase chain reaction
ERIC: Enterobacterial Repetitive Intergenic Consensus
UPGMA: Unweighted Pair Group Method with Arithmetic Mean
