# Supplementary figures and images for: The overlap of accessory virulence factors and multidrug resistance among clinical and surveillance Klebsiella pneumoniae isolates from a neonatal intensive care unit in Nepal: a single-centre experience in a resource-limited setting
Source: Trop Med Health. 2024 Apr 8;52:30. doi: 10.1186/s41182-024-00595-3 (PMC11000294; doi:10.1186/s41182-024-00595-3)

A)

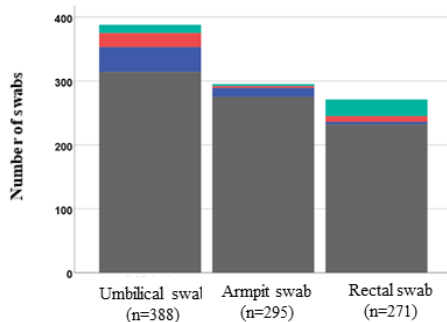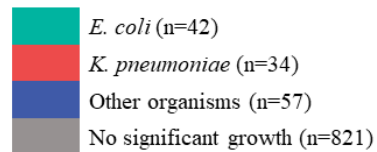

B)

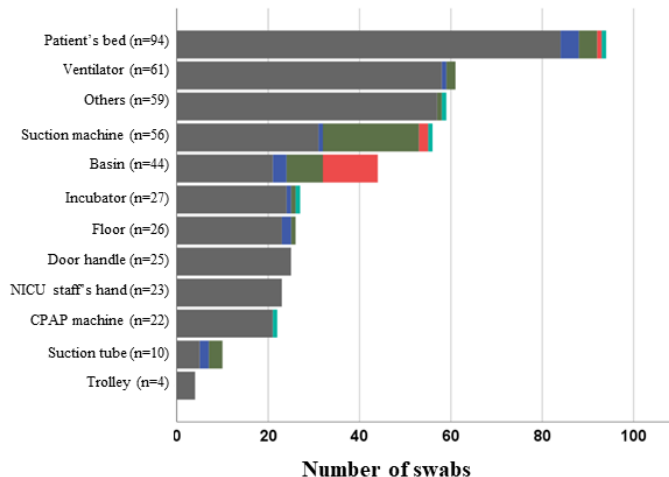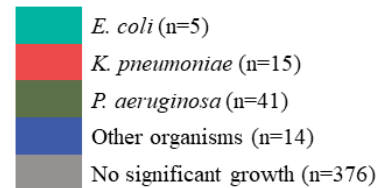

Supplement: Supplementary file 1 — Additional file 1: Figure S1. Results of A) neonate surveillance swabs and B) environmental surveillance swab cultures at the NICU of SMH within the study duration showing K. pneumoniae as one of the commonly isolated pathogens. [file 41182_2024_595_MOESM1_ESM.pdf]

# Virulence genes

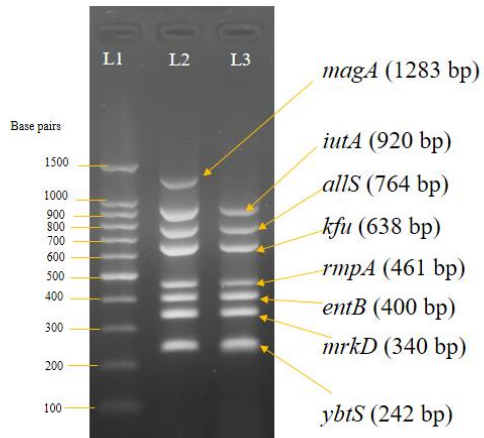

## *bla*<sub>CTX-M</sub>

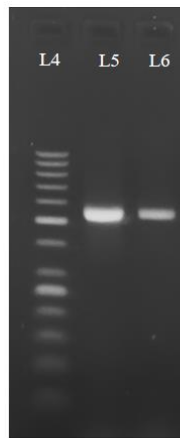

## *bla*<sub>TEM</sub> and *bla*<sub>SHV</sub>

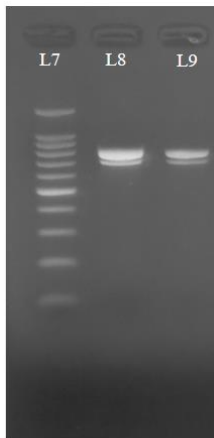

# pAmpC

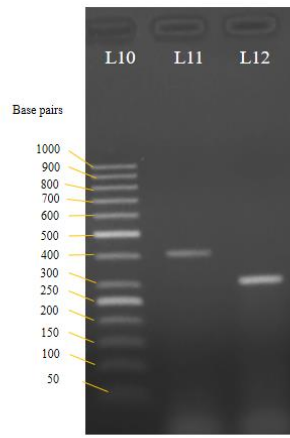

# Carbapenemases

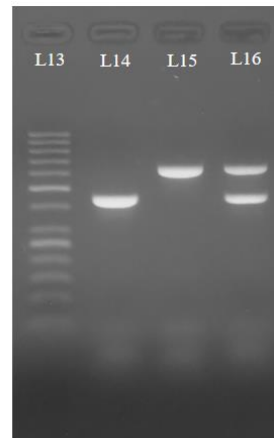

Supplement: Supplementary file 2 — Additional file 2: Figure S2. Gel electrophoresis results of PCR amplification products of representative samples showing genes investigated in this study. [file 41182_2024_595_MOESM2_ESM.pdf]

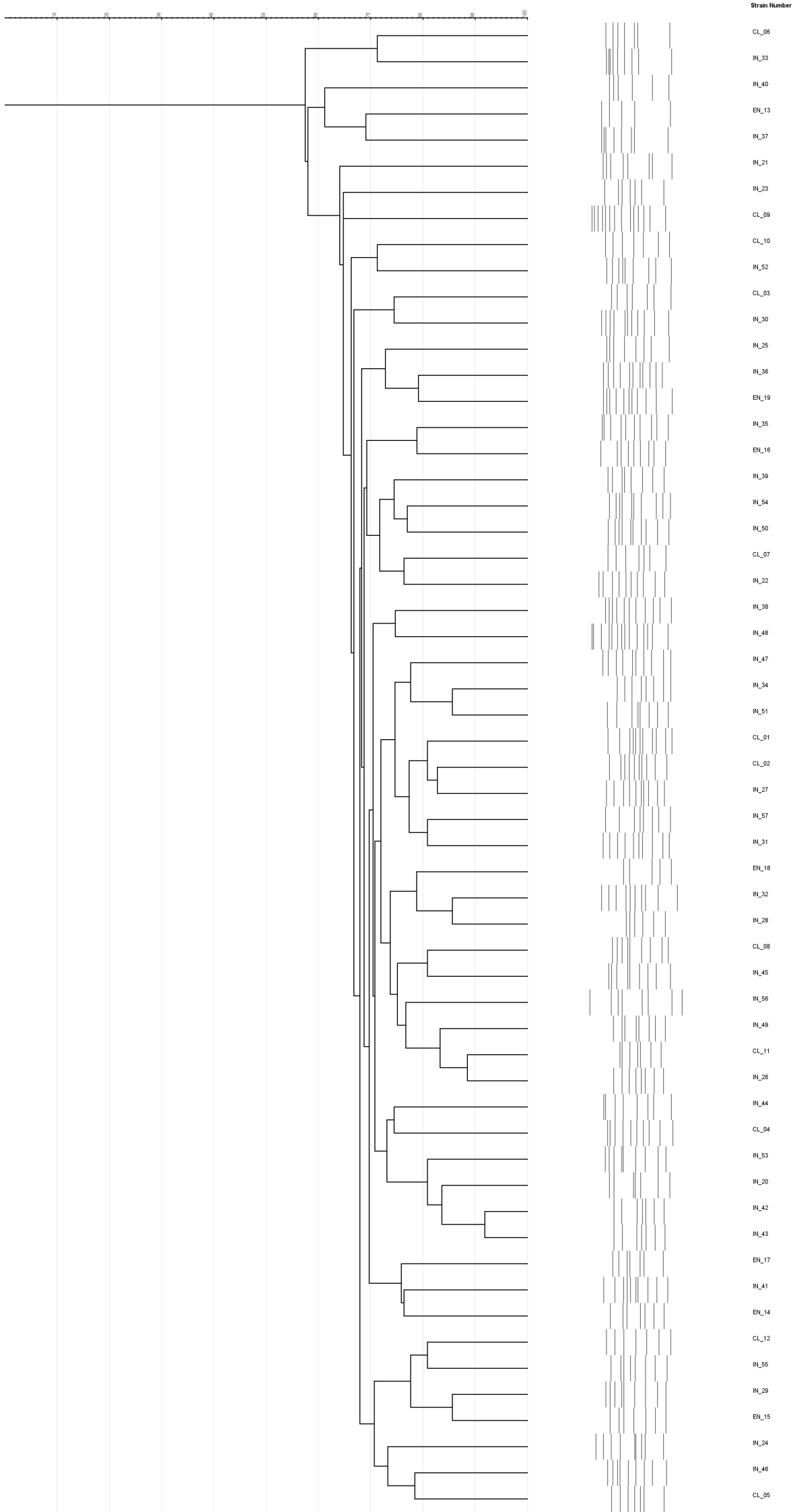

Supplement: Supplementary file 3 — Additional file 3: Figure S3. Dendrogram of ERIC–PCR fingerprints of 57 K. pneumoniae isolates based on Dice similarity and UPGMA linkage showing significant genetic heterogeneity among the isolates. [file 41182_2024_595_MOESM3_ESM.pdf]
