# Supplementary material for: The overlap of accessory virulence factors and multidrug resistance among clinical and surveillance Klebsiella pneumoniae isolates from a neonatal intensive care unit in Nepal: a single-centre experience in a resource-limited setting
Source: Trop Med Health. 2024 Apr 8;52:30. doi: 10.1186/s41182-024-00595-3 (PMC11000294; doi:10.1186/s41182-024-00595-3)
Supplement: Supplementary file 4 — Additional file 4: Table S1. Antimicrobial non-susceptibility patterns of infection-causing, possibly infection-causing, colonizing, and environmental K. pneumoniae isolates. [file 41182_2024_595_MOESM4_ESM.docx]

**Supplementary Table 1.** **Antimicrobial non-susceptibility patterns of infection-causing, possibly infection-causing, colonizing, and environmental *K. pneumoniae* isolates**

| **Type of isolates** | **Antimicrobials tested [No. of non-susceptible isolates (row percentage)]** | | | | | | | | | |
| --- | --- | --- | --- | --- | --- | --- | --- | --- | --- | --- |
|  | AUG | PTZ | CFM | CTX | CAZ | CPM | MEM | CIP | GM | AK |
| Infection (n=25) | 14 (56) | 8 (32) | 21 (84) | 21 (84) | 19 (76) | 14 (56) | 4 (16) | 16 (64) | 8 (32) | 7 (28) |
| Possible infection (n=13) | 4 (31) | 5 (38) | 9 (69) | 9 (69) | 9 (69) | 9 (69) | 2 (15) | 9 (69) | 4 (31) | 3 (23) |
| Colonizing (n=12) | 2 (17) | 4 (33) | 8 (67S) | 8 (67) | 7 (58) | 6 (50) | 2 (17) | 6 (50) | 2 (17) | 2 (17) |
| Environmental (n=7) | 3 (43) | 3 (43) | 4 (57) | 4 (57) | 4 (57) | 4 (57) | 3 (43) | 4 (57) | 3 (43) | 3 (43) |

Note: AUG: Amoxicillin-clavulanate; PTZ: Piperacillin-Tazobactam; CFM: Cefixime; CTX: Cefotaxime; CAZ: Ceftazidime; CPM: Cefepime; MEM: Meropenem; CIP: Ciprofloxacin; GM: Gentamicin; AK: Amikacin
