# Supplementary material for: The overlap of accessory virulence factors and multidrug resistance among clinical and surveillance Klebsiella pneumoniae isolates from a neonatal intensive care unit in Nepal: a single-centre experience in a resource-limited setting
Source: Trop Med Health. 2024 Apr 8;52:30. doi: 10.1186/s41182-024-00595-3 (PMC11000294; doi:10.1186/s41182-024-00595-3)
Supplement: Supplementary file 5 — Additional file 5: Table S2. Distribution of β-lactamases stratified number of accessory virulence genes among infection-causing, colonizing, and environmental isolates. [file 41182_2024_595_MOESM5_ESM.docx]

**Supplementary Table 2: Distribution of β-lactamases stratified number of accessory virulence genes among infection-causing, colonizing, and environmental isolates**

| **Type of isolates** | **No. of accessory virulence genes** | **ESBL gene or** *bla*_TEM_ | **pAmpC β-lactamase** | **Carbapenemases** |
| --- | --- | --- | --- | --- |
| **Infection-causing (n=25)** | |  |  |  |
| n=3 | 0 |  | | |
| n=1 | 0 |  | *bla*_DHA_ *bla*_EBC_ |  |
| n=6 | 0 | *bla*_CTX-M_ |  |  |
| n=1 | 0 | *bla*_CTX-M_ |  | *bla*_NDM_ *bla*_OXA-48_ |
| n=1 | 0 | *bla*_CTX-M_ |  | *bla*_OXA-48_ |
| n=3 | 1 |  | | |
| n=2 | 1 |  | *bla*_EBC_ |  |
| n=4 | 1 | *bla*_CTX-M_ |  |  |
| n=1 | 1 | *bla*_CTX-M_ *bla*_TEM_ |  |  |
| n=1 | 2 |  |  |  |
| n=2 | 2 | *bla*_CTX-M_ *bla*_TEM_ |  |  |
| **Associated with possible infection (n=13)** | |  |  |  |
| n=1 | 0 |  | | |
| n=1 | 0 | *bla*_CTX-M_ |  |  |
| n=1 | 1 | *bla*_CTX-M_ |  |  |
| n=2 | 2 |  | | |
| n=2 | 2 | *bla*_CTX-M_ |  |  |
| n=2 | 2 | *bla*_CTX-M_ |  | *bla*_NDM_ *bla*_OXA-48_ |
| n=2 | 2 | *bla*_CTX-M_ *bla*_TEM_ |  |  |
| n=1 | 3 | *bla*_CTX-M_ *bla*_TEM_ |  |  |
| n=1 | 5 |  |  |  |
| **Colonizing (n=12)** | |  |  |  |
| n=2 | 0 |  | | |
| n=1 | 0 |  |  | *bla*_NDM_ |
| n=1 | 0 | *bla*_CTX-M_ |  | *bla*_NDM_ *bla*_OXA-48_ |
| n=2 | 1 |  | | |
| n=2 | 2 | *bla*_CTX-M_ *bla*_TEM_ |  |  |
| n=1 | 2 |  | | |
| n=2 | 3 | *bla*_CTX-M_ *bla*_TEM_ |  |  |
| n=1 | 5 |  | | |
| **Environmental (n=7)** | |  |  |  |
| n=2 | 0 |  | | |
| n=1 | 0 |  |  | *bla*_NDM_ |
| n=1 | 0 |  |  | *bla*_NDM_ *bla*_OXA-48_ |
| n=1 | 1 |  | | |
| n=1 | 2 | *bla*_CTX-M_ |  | *bla*_NDM_ |
| n=1 | 2 | *bla*_CTX-M_ *bla*_TEM_ |  |  |
