# Supplementary material for: The overlap of accessory virulence factors and multidrug resistance among clinical and surveillance Klebsiella pneumoniae isolates from a neonatal intensive care unit in Nepal: a single-centre experience in a resource-limited setting
Source: Trop Med Health. 2024 Apr 8;52:30. doi: 10.1186/s41182-024-00595-3 (PMC11000294; doi:10.1186/s41182-024-00595-3)
Supplement: Supplementary file 6 — Additional file 6: Text. Additional details on the laboratory methods. [file 41182_2024_595_MOESM6_ESM.docx]

**Additional file 6: Additional details on the laboratory methods**

Phenotypic tests and DNA extraction were carried out at Siddhi Memorial Hospital’s microbiology laboratory and the molecular investigations were conducted at Kathmandu Center for Genomics and Research Laboratory, Lalitpur, Nepal. Siddhi Memorial Hospital (SMH) conducts internal quality control but does not have accreditation.

1. **General laboratory procedures at SMH**

Blood culture is conducted for neonates suspected of infection based on clinical symptoms as per the discretion of consulting paediatricians. About ≥1 mL blood sample is inoculated in a BD BACTEC^TM^ Peds Plus^TM^ bottle and incubated aerobically at 37 ^o^C. Turbidity is inspected every morning for up to 5 days. If turbidity is noted, the broth is inoculated into a 5% sheep blood agar and MacConkey agar and incubated aerobically at 37 ^o^C for 18-24 hours.

For NICU surveillance, swab samples from the admitted neonates and environmental swabs are collected. For neonates, umbilical, armpit, and rectal swabs are collected on the day of admission. Sampling sites and frequencies are based on SMH’s active infection prevention and control (IPC) team’s discretion. Due to resource limitations, environmental swabs are collected at least once every two months. When pathogens of concern (ESBLs, carbapenem non-susceptible pathogens, and MRSA) are isolated from admitted neonates or an outbreak is suspected or when patient density is high at a given time, environmental surveillance was conducted regardless of when the last sampling was done. All the swabs were plated directly into 5% sheep blood agar and MacConkey agar and incubated aerobically for 18-24 hours at 37 ^o^C.

1. **Identification and antimicrobial susceptibility**

If growth is observed, the isolate is identified by taking into account colony morphology, Gram’s straining, and biochemical profiling [catalase, oxidase, Triple sugar iron agar test, urease, SIM (Sulphur-Indole-Motility) test, and Simmon’s citrate] for Gram-negative bacteria. For this study, antimicrobial susceptibility testing (AST) was re-performed by Kirby Bauer disk diffusion, and susceptibility results were interpreted by CLSI guidelines (32^nd^ edition, 2022). Antibiotic discs and Muller Hinton agar were purchased from MAST. A calibrated densitometer (Grant Instruments, UK) was used for preparing 0.5 McFarland turbidity for performing AST.

*K. pneumoniae* isolates displaying non-susceptibility to any of the β-lactams were investigated by the D72C test (MAST, UK). Procedure and interpretation were done as per the manufacturer’s recommendations. Isolate identified as suspected carbapenemase producers was further tested by the modified carbapenem inactivation method (mCIM) in compliance with CLSI’s guideline to confirm the production of carbapenemases.

All the tests were subjected to quality control by *Escherichia coli* ATCC 25922. For the D72C test and mCIM, additionally, a clinical isolate previously confirmed to harbor *bla*_NDM_ was used.

1. **Molecular investigations**

Crude DNA was isolated for all isolates by heat lysis method. Briefly, a few well-isolated colonies from Sheep blood agar were picked and inoculated into 500 µl of nuclease-free water to prepare a smooth suspension of the colonies. The suspension was vortexed and boiled for 20 minutes at 80 ^o^C. Then, the sample was centrifuged at 13,500 rpm for 5 minutes and the supernatant was preserved at -40 ^o^C until further use.
